# Clinician acceptability of an antibiotic prescribing knowledge support system for primary care: a mixed-method evaluation of features and context

**DOI:** 10.1186/s12913-023-09239-4

**Published:** 2023-04-14

**Authors:** Ruth Hurley, Francine Jury, Tjeerd P. van Staa, Victoria Palin, Christopher J. Armitage

**Affiliations:** 1 Manchester Centre for Health Psychology, Division of Psychology and Mental Health, School of Health Sciences, Faculty of Biology, Medicine and Health, The University of Manchester, Manchester, UK; 2 Centre for Health Informatics, Division of Informatics, Imaging and Data Science, School of Health Sciences, Faculty of Biology, Medicine and Health, The University of Manchester, Manchester, UK; 3 Manchester Centre for Health Psychology, Faculty of Biology, Medicine and Health, Division of Psychology and Mental Health, School of Health Sciences, The University of Manchester, Manchester, UK; 4 Academic Health Science Centre, Manchester University NHS Foundation Trust (MFT), NIHR Greater Manchester Patient Safety Translational Research Centre, The University of Manchester, Manchester, UK

**Keywords:** eHealth, Intervention, Qualitative, Mixed-method, Acceptability, Co-design, Knowledge-based support, Decision support systems, User-focused design

## Abstract

**Background:**

Overprescribing of antibiotics is a major concern as it contributes to antimicrobial resistance. Research has found highly variable antibiotic prescribing in (UK) primary care, and to support more effective stewardship, the BRIT Project (Building Rapid Interventions to optimise prescribing) is implementing an eHealth Knowledge Support System. This will provide unique individualised analytics information to clinicians and patients at the point of care. The objective of the current study was to gauge the acceptability of the system to prescribing healthcare professionals and highlight factors to maximise intervention uptake.

**Methods:**

Two mixed-method co-design workshops were held online with primary care prescribing healthcare professionals (*n* = 16). Usefulness ratings of example features were collected using online polls and online whiteboards. Verbal discussion and textual comments were analysed thematically using inductive (participant-centred) and deductive perspectives (using the Theoretical Framework of Acceptability).

**Results:**

Hierarchical thematic coding generated three overarching themes relevant to intervention use and development. Clinician c*oncerns* (focal issues) were safe prescribing, accessible information, autonomy, avoiding duplication, technical issues and time. *Requirements* were ease and efficiency of use, integration of systems, patient-centeredness, personalisation, and training. Important *features* of the system included extraction of pertinent information from patient records (such as antibiotic prescribing history), recommended actions, personalised treatment, risk indicators and electronic patient communication leaflets. Anticipated acceptability and intention to use the knowledge support system was moderate to high. Time was identified as a focal cost/ burden, but this would be outweighed if the system improved patient outcomes and increased prescribing confidence.

**Conclusion:**

Clinicians anticipate that an eHealth knowledge support system will be a useful and acceptable way to optimise antibiotic prescribing at the point of care. The mixed method workshop highlighted issues to assist person-centred eHealth intervention development, such as the value of communicating patient outcomes. Important features were identified including the ability to efficiently extract and summarise pertinent information from the patient records, provide explainable and transparent risk information, and personalised information to support patient communication. The Theoretical Framework of Acceptability enabled structured, theoretically sound feedback and creation of a profile to benchmark future evaluations. This may encourage a consistent user-focused approach to guide future eHealth intervention development.

**Supplementary Information:**

The online version contains supplementary material available at 10.1186/s12913-023-09239-4.

## Introduction

Antimicrobial resistance (AmR) is a global health concern driven by overuse of antibiotics in agriculture and medicine [1]. In England 84% of antibiotics are prescribed in primary care and despite a 23% reduction in prescribing in the last five years, cases of antimicrobial resistance are still a prescient concern [2]. There remains substantial variability in antibiotic prescribing to primary care patients with common infections and a lack of risk-based prescribing such that there is no association between prescribing decisions and patients’ risk of infection-related complications [3-5]. Comprehensive Clinical Practice Research Datalink (CPRD GOLD [6]) investigations of longitudinal primary care prescribing patterns 2012 to 2017 found that more consistent prescribing based on patient type (case mix) and patient risk of complications could substantially reduce antibiotic prescribing. A personalised patient-risk-based approach would therefore help to reduce opportunities for antibiotic resistance while ensuring that patients who most need antibiotics get them [7, 8]. Feedback dashboards and reports have been developed that allow practices and individual clinicians to monitor trends in their antibiotic prescribing periodically [9, 10]. However, clinicians may also benefit from structured information about individual patients to inform decision-making at the point of care. A Knowledge Support system (KS) is being implemented to fulfil this requirement as an eHealth programme combining medical knowledge and AI to support clinicians during consultations to make their own evidence-based decisions [11, 12]; see Supplementary file 1 for further information). By linking research and clinical practice, the KS takes steps toward a learning health system approach by allowing continuous improvement in care models [13, 14]. The KS will be tested in a large randomised controlled trial [7].

Changing GP behaviour (such as prescribing) is recognised as a challenge [8, 15-18] being influenced by multiple capabilities, opportunities and motivations (COM-B; [19]. Clinical decision support systems (CDSS) can be effective in supporting prescriber behaviour change [20, 21], however numerous meta-analyses and systematic reviews show they are not widely used [22] and have inconsistent outcomes [23-25]. Key criticisms include poor documentation of behaviour change strategies, a resulting failure to learn the lessons from past interventions [17, 26-30], and poor attention to system design and implementation features. More research on eHealth success factors have been called for [31] but Medical Research Council guidelines point to a lack of consideration of user focus and acceptability [32, 33].

User evaluations of computer-based tools often focus on narrow measures of acceptability that predict system use (such as intention or usage statistics; [34] and so one of the aims of the present study was to use the Theoretical Framework of Acceptability (TFA; Sekhon et al., 2017, 2018) to conduct a comprehensive assessment of acceptability. According to the TFA, there are seven key dimensions of acceptability, namely: *affect* (feelings), *burden* (effortful aspects), *perceived effectiveness* (whether it will fulfil its purpose), *ethicality* (fit with personal values), *coherence* (whether the intervention makes sense and can be understood), *opportunity costs* (things that will have to be given up to use the intervention), and *self-efficacy* (confidence in their ability to use the intervention). Additionally, questions derived from the framework can be used to judge anticipated acceptability which is useful to help refine intervention design at an early prototype stage [35, 36]. The current research reports on a qualitative study of interactive online stakeholder workshops. The research objectives were to obtain feedback to inform participative design of a person-centred KS, including (1) usefulness of suggested features (including individualised feedback for GP and associated patient leaflet), (2) anticipated acceptability and intention to use the system, and (3) contextual factors that may affect clinician uptake.

## Method

Two mixed-method online workshops were held in July 2021 with antibiotic prescribing healthcare professionals working in primary care in England. The convergent QUAL-QUAN design combined interactive co-design focus groups with (1) formative evaluation of suggested features, (2) summative evaluation of anticipated acceptability and intention to use the system (3) interpretive consideration of acceptability and contextual issues. The topic guide (see Supplementary file 2) incorporated perspectives in epidemiology and health intervention development, stakeholder engagement, e-learning resource development, psychology, behaviour change and implementation science.

### Recruitment and sampling

Primary care health professionals who prescribe antibiotics were purposively recruited via event links sent to professional network practitioner groups, and surgeries. Participants were compensated for their time with a £100 shopping voucher.

### Measures

**Fig. 1:**
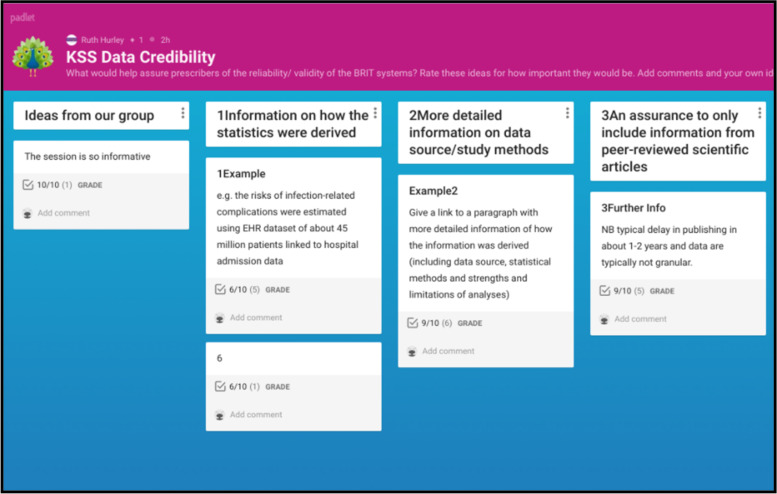
Screenshot of the online Padlet used to rate KS example features

*Online collaboration whiteboards* (Padlet v153.0; see Fig. 1) to evaluate (i) example features, (ii) effectiveness of existing patient communication methods (0 = not useful, 10 = very useful), and (iii) KS credibility features (0 = not important, 10 = very important). Clinician engagement (number of ratings) was also noted. Examples (including patient risk information, prescriber feedback messages, recommended actions and caution messages) were derived by the development team based on previous BRIT project research and anticipated capabilities of the KS [9].

**Fig. 2:**
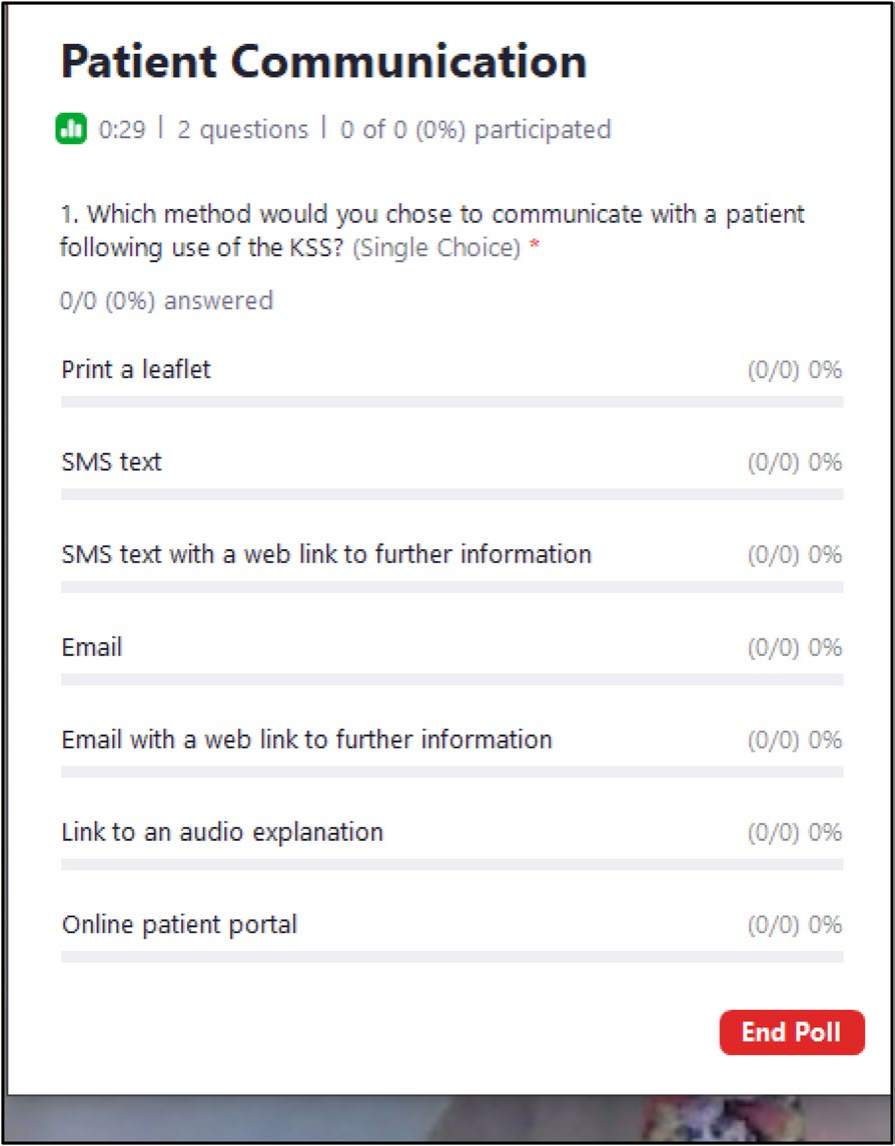
Screenshot of a video conference polling question: Summative feedback on patient communication

*Online Zoom polls* (Zoom Video Communications; see Fig. 2) were used to gather summative data on preferred methods for (i) opening the KS (ii) patient communication, and workshop evaluation.

*Acceptability and intention to use the KS* were rated using an online survey (Qualtrics XM, Utah, USA v062021; see Supplementary file 3) using the seven domains of the Theoretical Framework of Acceptability [35, 37] TFA; on a scale from 0–10 with optional open text comments. To examine convergent validity, ratings for intention, expectation and desire to use the KS [38] were also captured (0 = very negative, to 10 = very positive).

### Data collection

**Fig. 3:**
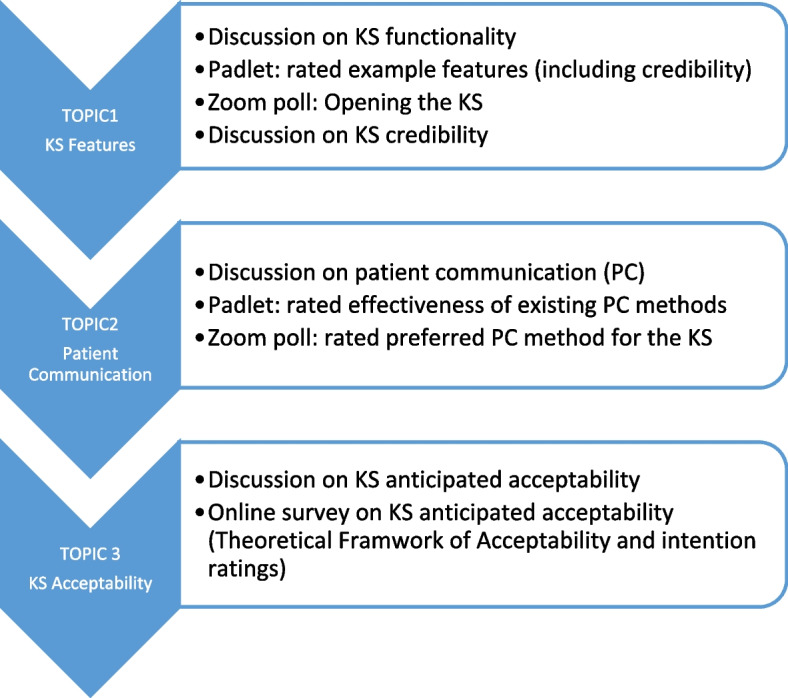
Workshop topic guide and data collection methods

Each 220-min workshop explored KS features, patient communication and KS acceptability incorporating multiple modes of interaction and response for flexibility (see Fig. 3). Two members of the research team experienced in stakeholder research (RH and FJ) acted as facilitators and led a discussion encouraging verbal and text comments. Online whiteboards (Padlets, see Fig. 1) were used to provide participants with example features that they could all simultaneously rate and comment on. Online polls were used to get an accurate snapshot of participants’ views. Anticipated acceptability of the proposed KS was discussed using questions derived from the TFA and participants accessed an online survey to rate acceptability, intention, optional comments, and demographics (see Supplementary file 4 for data sources).

### Data analyses

Qualitative data (including the audio transcript, chat files and comments) were analysed within NVIVO (QSR International,v12 +) following the Framework approach deemed appropriate for interdisciplinary mixed-method health research data [39]. After transcription and immersion first order (inductive, descriptive) coding used directed content analysis [40] to highlight points of interest in the text with a first person perspective. Second Order (inductive, interpretive coding) explored how first order codes were being discussed. A sample of each unit of analysis (Zoom chat, survey comments, and verbal discussion) was independently coded and discussion between coders followed to agree relevant descriptive categories. Themes and sub-themes were formed hierarchically by grouping codes according to meaning, similarities and differences. Importance was assessed based on frequency and intensity of expression; the most prominent categories met both criteria (consistent with Keyworth et al., 2019). Third Order (deductive coding) was used to consider the second order codes in relation to the relevant domains of the TFA and intention to use the KS. Quotes representative of the data corpus were selected. The full hierarchical list of themes, codes and quotes were summarised in a code book and interpreted in memos, drawing out implicit themes in relation to antibiotic prescribing, eHealth systems and features of the KS including patient communication. Quantitative findings (feature usability ratings, engagement, and acceptability) are summarised and discussed here-in alongside the relevant qualitative insights with reference to JARS-Qualitative reporting standards [41].

### Ethics

Ethical approval for the study was obtained from the North East—Newcastle & North Tyneside 2 Research Ethics Committee Ref 21/NE/0103. Participants returned online consent forms before entering the session, and provisions for withdrawal were re-stated at the start of the session [42].

## Results

### Participants

Clinicians (*n* = 16) had a range of medical experience from general practitioners, to prescribing nurses (see Table 1).

**Table 1:** Participant demographics (total *n* = 16)

**Job Role**	n
GP	4
Advanced Nurse Practitioner	2
Practice Nurse	1
Practice Pharmacist	3
Pharmacist	4
Other Administrative (non-prescribing)	2
Preferred not to say	2
**Ethnicity**	
White	7
Asian or British Asian	1
Other	2
Black or Black British	0
Preferred not to say	4
**Gender**	
Female	14
Male	2
Preferred not to say	0
**Age**	
Mean	46
Range	39 to 65
Preferred not to say	1

### Mixed-method analysis: qualitative and quantitative findings by theme

Key points from the qualitative and quantitative analysis are presented under the four overarching themes of concerns, requirements, KS features and KS acceptability (see Tables 2, 4 and 7 for sub themes and indicative quotes).

**Table 2:** Summary of primary and secondary themes and indicative quotes for clinician concerns and requirements

**1º Themes**	**Concerns**	**Requirements**
**2º Sub-themes**	**Safe and accurate prescribing** *‘…Major part of it, would be to make sure I’m not prescribing something incorrectly’*	**Clarity of content and purpose** *‘Step by step explanation of what it is, how it benefits us, and what the risk score means’*
	**Information accessibility** *‘looking back on records opening up documents seeing what's come before so, especially because prescriptions from urgent care any clinics, they will not appear on a medication screen, so they take time to look for.’*	**Ease and efficiency of use** *‘So I think, really, we have to think people, especially my generation, you know we weren't brought up with all this it so it has to be easy to use, it really does.’*
	**Patients**^**1**^ *‘At the end of the day, you want to do the best by your patient …helping the patient out through avoiding incorrect prescribing’*	**Integration/ Interoperability** *‘I was kind of in agreement with what everyone was saying about moving in between systems and just making sure that it was all kind of concise, to the point that still have the relevant information on there’*
	**Avoiding duplication** *‘Must have additional benefit to current systems or it won’t get used.’*	**Patient-centred** *‘Ensuring my patient understands the advice and treatment offered is vital to me.’*
	**Time** *‘Because sometimes you're at a lost end and you can't think because they're allergic to so many things, and you can't think what you're meant to do… anything that will save us [time] and be safer.’*	**Personalised** *‘Personalised ones [leaflets] I think will go down well as patients might feel they’re being ‘fobbed’ off with generic info’*
	**Technical issues** *‘Because in a 10 min consultation with the patient you don't want to be struggling with a template which you know takes us time opening up or that we have to search through.’*	**Training and documentation** *‘IF given proper training I think it will be very useful… I find that training is often lacking.’*
	**Autonomy** ^**2**^ *‘I would prefer something that you could actually open up yourself, when you feel a bit… out your depth or [the case is] a bit … difficult.’* *‘Would we open that up if it wasn't automatic? I mean if it's an automatic opening we can we can shut it down if we don't want to see it?’*	**Accessibility**^**3**^ *‘so that we could see it in their language and in English and know exactly what the advice they're being given is, and then we can sort of comment and clarify’*

Tertiary sub-themes: 1 = Patient Care, Communication, Reactions; 2 = Autonomy Freedom of choice, Reliance on tools; 3 = IT-literacy and access, Language barriers, Learning Difficulties

#### Thematic Analysis: Concerns and requirements themes and indicative quotes

##### *Concerns: what clinicians told us*

Clinician concerns were defined as problematic or focal issues emphasised by participants (regarding antibiotic prescribing and use of computer systems to support this). Participants’ priority concern was safe prescribing that accurately addresses patient health issues (preventing harm, repeat consultations and dissatisfaction). Clinicians were also concerned about the potential for duplication of effort and of system functionality which would waste valuable time, add cognitive burden and cause annoyance. The way information was displayed in the electronic health record (EHR) was a concern as it did not always support fast decision-making incorporating different streams of information, for example, the ability to prescribe and view patient data simultaneously. It was not feasible to take time to search through the EHR in the consultation, so key details and opportunities for patient education were being missed. Not all clinicians felt confident with technology and there was a concern about any system that was too slow or might lead them to struggle in front of a patient. A final recurrent issue was autonomy and the desire (especially emphasised by experienced GPs) to be able to choose how and when eHealth tools were used, however all participants appeared to welcome some level of antibiotic prescribing support, especially in respect of selecting safe appropriate medication for complex cases.

##### *Requirements: what clinicians told us*

Clinician concerns, issues, and experiences fed into six broad requirements for eHealth computer systems. *Ease and efficiency of use* was a top priority. It was very clear that if the system was too complex or time consuming it would not be used. Most clinicians agreed that time to use the KS should be less than a minute. The system should also be accessible for clinicians with disabilities and learning difficulties. Participants needed the system to *work cohesively with their EHR* so they could view the information they needed concurrently. The system also needed to offer more than generic guidelines or warnings that can already be accessed through the EHR. System functionality needed to be centred on the *best health outcomes for the patient* including *personalised knowledge* relevant to the patients’ medical history, treatment and local AmR priorities (going beyond general national guidelines) which would be genuinely useful and lead to more informed prescribing decisions. Effective *training and documentation* (stated to be often lacking), plus clarity of KS content and objectives were also very important to encouraging ‘buy-in’ from colleagues.

##### *KS Features*

Clinicians were presented with examples of KS Features and were asked to rate them (see Tables 3 and 5). Qualitative analysis of features that clinicians wanted to see in the KS were summarised under themes of Inputs and Outputs (see Table 4 for sub themes and quotes).

**Table 3:**
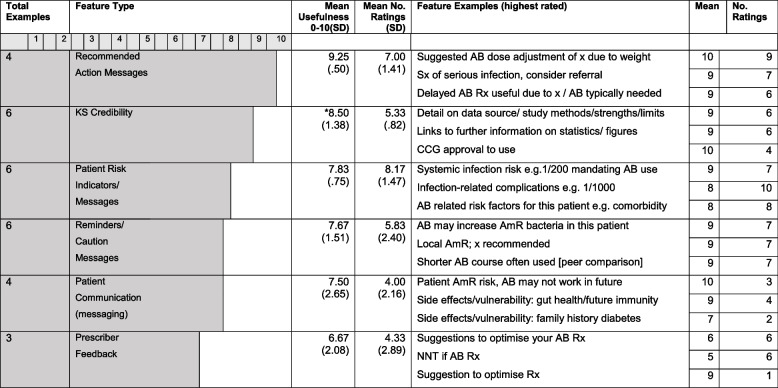
KS Example features summarised by feature type: mean usefulness ratings and mean number of ratings (engagement)

*AB* Antibiotics, *AmR* Antimicrobial Resistance, *CCG* Clinical Commissioning Group, *NNT* Number Needed to Treat, *Rx* Prescribing, * = Importance rating

**Table 4:** Thematic analysis: KS features sub themes (primary, secondary, tertiary) and indicative quotes

**1º Theme**	**KS Features**	
**2º Themes**	**Inputs**	**Outputs**
**3º Themes**	**Existing Patient Data** *“Any system that will just you click on a link, like the sign one and it brings everything in, and it will tell you in in seconds”*	**AB Rx Summary & Information** *‘No but that IS useful… it would be very good to have all this data on one page without having to move between screens.’* *‘Susceptibility to severe infection (e.g. diabetes), previous hospitalisation for similar infection, last antibiotics given (name and date), antibiotics allergies’*
	**Search Tools** *“If a list of appropriate medication is presented after diagnosis, I would click to view a range of options of meds to prescribe for a choice of agent.”*	**Recommended Actions** *‘What abx for that indication could be prescribed. Then, choosing from a list with a link to current medications that the patient is already on e.g. not a macrolide if on anti-psychotics QT interval or patient pregnant avoid X drug at term—linking in with how many weeks pregnant pt. is* *‘…we ended up spending a load of time speaking to the ENT registrar for advice about further possible AB to give … and it would have been very helpful … get advice about you've tried this you've tried this, now, this is the next step.’*
	**Templates** *‘you click the bring up the… hearing template the QRisk scoring templates and this recently is a COVID review template that is been coming up so similar things similar on those lines would be that helpful.’*	**Credibility** *‘The information about CCG endorsement, research and trials would help.’*
	**KS Opening** *‘So in the diagnosis I’d fill in a read code … so I would put it in at ‘Diagnosis’ and then use the use the tool at that point, and then at the bottom put the plan from the tool, or you know, using the support of the tool.’*	**[Avoid] Popup Notifications** *‘The pop-ups can get a bit tedious sometimes you end up closing them.’*
	**Reception Triage** *‘I get it, it will help… in some instances they might be able to get it, but I don't know I think it's a lot of responsibility.’*	**Patient Communication** *‘Ability to text pt. [patient]. If they have mobile; Text or phone call—remote consultation (EMIS user); AccuRx text is most useful. You can send links for leaflets, other info and option for them to text back too’* *‘and to help them understand their personal relationship to the antibiotics, so perhaps any risks that they've got from the antibiotic or maybe the fact that they've already had [an antibiotic]’*

### Example KS features: clinician ratings

Qualitative ratings of example features, summarised by feature type, mean usefulness and mean number of ratings (engagement).

*Recommended Action* messages were rated as the most useful type of KS feature (see Table 3), including suggested dose adjustments (due to weight or renal function), flags when a patient displayed symptoms of severe or systemic infection (indicating referral), and when it was appropriate to consider a delayed antibiotic. Participants discussed that recommendations around treatment would be useful, especially in terms of what to try next when patients re-consult, for example because initial antibiotics did not work.

*KS Credibility* was important to clinicians including NICE and CCG endorsement. Having further information and the assurance of use of peer-reviewed data were rated as important by most (although an alternative view was that credibility would be self-evidential based on practice approval). Participants were enthused about the idea of *Patient risk* indicator scores similar to outputs generated from templates like QRisk [43] to inform antibiotic prescribing. *Prescriber feedback* features had lower usefulness ratings and engagement than other examples, possibly due to high patient focus and time-pressure within the consultation context.

### KS Features: what clinicians told us

#### KS Inputs

Clinician discussion and poll data (70%; *n* = 9) showed a clear preference to be able to *open the system manually* from a button on their EHR tool bar. The preference was chiefly motivated by alert fatigue but also a desire to use the KS mainly for more complex cases (especially from more experienced prescribers). It was recognised that automatic opening could be a useful prompt at times. It was very important (for accessibility, usability and time constraints) that data would be drawn from the EHR and written back automatically wherever possible, *minimising data input*.

#### KS Outputs

Clinicians needed clear concise *summaries of patient information* to support their treatment/prescribing decisions. Summaries should include relevant patient conditions, previous antibiotics, previous hospitalisation, suitable antibiotics recommendations that considered interactions with existing medication and co-morbid conditions.

Clinicians discussed the significance of *making sure patients understood* their prescribing decision emphasising the importance effective of patient communication. This included relevant dialogue and the ability to give (or send) information to digest after the consultation. Content that communicated the risk of antibiotic resistance, and ‘patients’ individual vulnerabilities and side effects’ were rated highly (particularly in relation to repeated antibiotic use) but ‘patient risk of developing serious complications compared to other patients of a similar age/ gender’ attracted a low mean rating (see Table 3).

##### Patient communication methods: Clinician ratings

Effectiveness of existing patient communication methods and preferred methods for the KS were rated (see Table 5). Text messages (with hyperlinks to further information) were rated 10/10 for effectiveness and were the preferred method for the KS chosen by 55% of respondents (6 out of 11 poll responses). Two respondents found patient portals effective, but these seemed less widely used. Electronic patient communication was more prevalent due to COVID-19, but paper leaflets (9/10 for current effectiveness) were also valued (especially for patients without devices). Accessibility options to consider included low literacy, different languages, sight impairment and digital inequalities.

**Table 5:** Preferred methods of patient communication for the KS (effectiveness ratings, comments and frequency chosen as the preferred KS method by Zoom poll responses)

**Method**	**Mean Effective-ness Rating(/10)**	**No. of Ratings**	**Frequency of preference (Zoom Poll)**	**Comments about the method (Padlet)**
**Text message**	10.00	6	6	*“Ability to text pt. if they have mobile”;* *“Text or phone call—remote consultation(EMIS user)”;* *“AccuRx *[44]* text is most useful. You can send links for leaflets, other Information/Example and option for them to text back too.”*
**Dialogue with the patient**	10.00	4	NA	
**Printing a Leaflet**	9.00	4	2	*“pts like this as they don’t remember everything you tell them”*
**Email**	6.00	3	1	*“Haven’t used email”*
**Patient portal**	5.00	3	2	

^*^Patient Communication Method ~ Poll Options (frequency chosen as the preferred method): 1 = Print a leaflet B&W(1); 2 = Print a leaflet colour(1); 3 = Simple prewritten text (SMS; 1); 4 = Text (SMS) prewritten with a hyperlink to a secure webpage(5); 5 = Simple prewritten email (0); 6 = Email with a hyperlink to a secure webpage (0); 7 = Email with a PDF attachment (1); 8 = Patient portal (2)

##### KS Acceptability

Anticipated acceptability of the KS was discussed and rated along the seven dimensions of the TFA. The mean ratings were used to construct a visual acceptability profile for the intervention, along with three intention ratings and indicative quotes (see Tables 6 and 7). The KS had high mean acceptability ratings for affect, ethicality, self-efficacy, and perceived effectiveness. ‘High’ was judged to be the top third (or 7.00 and over) in keeping with [45]. Burdens and costs were low to moderate (4.00–5.00) and coherence of the system was moderate (6.00). These areas should be monitored and potentially targeted for improvement. Participants’ mean intention to use the system was high on all three measures but highest for desire.

**Table 6:**
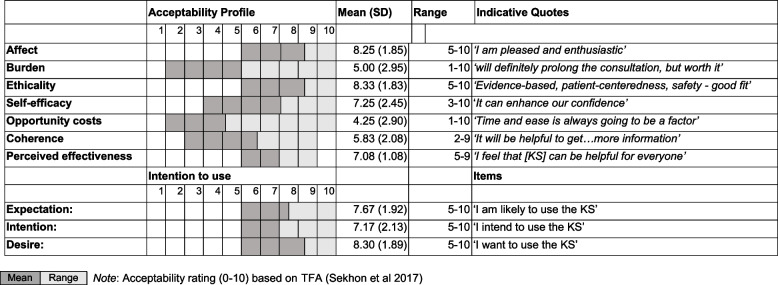
KS anticipated acceptability profile: mean and range of ratings and indicative quotes; *n* = 12)

**Table 7:** Thematic analysis: KS Acceptability (and intention) sub themes and indicative quotes

**1º Themes**	**KS Acceptability**
**2º Sub-themes**	**Affect** *‘I am pleased and enthusiastic as it will be easier for me to give information to my patients …’* *‘In principle it sounds like a good idea’*
	**Burden** *‘It is time, I mean we're … horrendously busy in GP practices.’* *‘If the system works at an acceptable speed not much; inputting data and then waiting for the result when busy makes me impatient.’*
	**Ethicality** *‘I would feel safer’* *‘Minimizing risks and giving as much information as possible is always a good thing. I feel that in the long run this will save time (not having to refer to others or make additional consultations that might take time, affecting the patient's wellbeing)'*
	**Self-efficacy** *‘Very confident’* *‘Difficult to be confident at this stage but I very much hope so!’* *‘More confidence and easy to access literature/ information that can support the decisions made’*
	**Opportunity costs** *‘The most that it'll take of us is time because if you're pressed for time with a 10 min consultation, even a few seconds, sometimes even half a minute can make the difference.’* *‘I guess we would be giving up our ability as clinicians to know… relying on a tool instead of having the knowledge to make those judgements ourselves.’*
	**Coherence** *‘A clearer message on what is trying to achieve. Its place in the prescribing of antibiotics, its relevance—are you looking to streamline NHSE antibiotic prescribing? Are you looking that all abx are reviewed after repeat courses? How are you monitoring inappropriate use?’* *‘More training, and good reference guide.’*
	**’Perceived effectiveness** *‘If it's easy to use and pulls in the data for me, EMIS or system one and that you don't have to input loads of data yeah and it will be very effective.’* *‘I feel that can be helpful for everyone. Especially for those patients that are on rescue packs of antibiotics that might end up not working. And to avoid errors in prescribing.’*
	**Intention** *‘Might be some teething problems like always with a new system but I feel it's worth trying.’* *‘it's really easy to say 'oh I’m not going to bother to use this'* *‘Happy to use’*

## Discussion

The current study combined evaluation of intervention features and acceptability with thematic analysis to support a user-focused approach to develop an antibiotic prescribing KS. Inductive and deductive thematic analysis identified core themes related to clinician concerns and requirements. Sub-themes within clinician c*oncerns* (focal issues) were safe prescribing, accessible information, autonomy, avoiding duplication, technical issues, and time. *Requirements* were ease and efficiency of use, integration of systems, patient-centeredness, personalisation, and training. The combined thematic and acceptability findings in the analysis were used to generate suggestions to make the KS more acceptable by reducing burdens, improving perceived efficacy and coherence of the tool.

Ratings and thematic analysis indicated moderate to high acceptability of the KS across multiple domains of the TFA [36] including positive feelings and compatibility with personal values (such as patient centeredness). The main effort and opportunity cost identified was time to learn and use the system. Combined insights from the analysis were used to generate design recommendations and visualisations to help communicate user requirements with the multi-disciplinary development team (see Supplementary files 5 and 6).

Lack of time was a recurrent theme in the analysis and a key anticipated cost of system use. Short consultation times are an established barrier to effective care, patient communication, and guideline adherence [46, 47] and have been shown to influence antibiotic prescribing. High antibiotic prescribers also tended to report being more risk averse and more affected by concerns about the clinical relationship [8, 18, 48]. The issue of consultation time and resource demands are heavily dependent on health policy; therefore, it is essential interventions must maximise clinician time (e.g., optimise cognitive processing) and support clinician’s confidence in their appraisal of patient risk.

A KS that is *easy to use* would reducing the likelihood of staff struggling with technology in front of a patient (a core concern) and support fast integrated workflow. Comprehensive *training and documentation* (including clear system aims) were identified by participants to reduce intervention burdens/costs (such as technical issues, time, and stress). This would be at its highest while clinicians were learning to use the system. Training supports self-efficacy, intervention coherence, increases persistence and reduces defensive processing [49] making it an important way to improve KS acceptability for clinicians who are least-likely to want to use the system [50], i.e. those whom we most want to encourage.

*Personalised*, locally relevant information [18] such as local guidelines, patient, practice, and prescriber insights are difficult to obtain quickly and therefore provide *unique functionality* which makes system use worth the time. In general, features that support autonomy like customisation appeared more motivating [33] while generic features like static/ generalised patient communication leaflets were deemed less useful.

### KS features

The workshops helped to gauge importance of KS features including extraction of pertinent information (such as antibiotic prescribing history) from patient records, recommended actions, personalised treatment, and risk indicators. Effective, personalised patient information was central to clinician values and policy in the form of patient understanding and shared decision making. Interventions that improve clinician communication with patients have been shown to improve clinician self-efficacy, intervention efficacy and patients’ experience of quality of care [51-53]. A meta-analysis of interventions to reduce antibiotic prescribing in general practice found shared decision-making interventions to be the most successful (along with point-of-care testing) but patient leaflets were only effective in reducing prescribing when used ‘interactively’ as part of the intervention and clinical discussion [28, 54]. Discussion prompts should be considered as an extra resource to support communication [55, 56].

### Strengths

The study had several strengths. In line with complex intervention development guidelines [32] acceptability measures were used to establish if there is a demand for the KS, and help identify ways to maximise uptake. The TFA was used as a framework to both code and rate emotional and cognitive acceptability along seven dimensions, providing a sound theoretical basis for the evaluation [35]. The positive acceptability evaluation was reinforced by the moderate to high ratings for clinicians’ expectation to use the KS which is a likely predictor of behaviour (being more strongly associated with self-efficacy, and more likely to reflect perceived barriers compared to similar constructs of intention, or desire [38, 57]. The online nature of the workshops was turned into an advantage and allowed innovative strategies to engage stakeholders, collect feedback and triangulate findings. Qualitative insights are essential to help identify trends and patterns to interpret complex phenomena [58] such as prescriber views and intervention feedback. Formative and summative measures (polls/surveys) allowed assessment of convergence (complementarity) of responses; [59]. Outputs from the analysis were directly used to improve and guide intervention design. Going forward the TFA framework also provides an opportunity to monitor the acceptability profile of the system as it develops, ensuring user-centred design is kept to the fore.

### Limitations

The study benefited from input from a range of primary care prescribers (general practitioners, advanced nurse practitioners, practice pharmacists), but were all interested and motivated to address AmR, and the group setting could have increased the opportunity for social desirability bias [60, 61]. Findings do resonate with issues identified in narrative, meta-analyses and systematic reviews of decision support tools [21, 22, 62-64]. The existing sample is sufficient to inform early content development, but KS would benefit from a larger sample to allow inferential testing of qualitative ratings and purposive sampling to a wider cross-section of participant views. As TFA scale cut-offs are still becoming established interpretation of ratings scores in the current study were conservative, interpreting high acceptability score as 7.00 or more. Previous studies have used 5.00 or more [37] but the efficacy of this cut point will only become apparent with more wide spread use of the framework. Future evaluations of the system would benefit from a wireframe or test system to help give participants a more coherent understanding of the intervention and gather views from users who may initially be less receptive to the intervention. Subsequent evaluations should compare the acceptability profile as the system is developed. It may also be useful to assess injunctive social influences of patients on KS use [65].

The TFA acceptability profile proved a useful replicable way to evaluate intervention acceptability and monitor ongoing person-centred development of the KS (including costs, burdens, and coherence). Wider exploration of clinician concerns gave additional context to the dimensions of acceptability including the additive effects of clinician concerns costs and burdens, for example lack of physical opportunity (time) amplified issues with psychological resources (cognitive overload, decision fatigue, lack of reflection time). Intervention developers should also consider the combined effects of dimensions of acceptability [26].

## Conclusion

Mixed-method online stakeholder workshops confirmed that there is a demand for explainable personalised patient risk information, accessible summaries of pertinent patient data and personalised literature to support patient-clinician communication in respect of antibiotic prescribing. Clinicians anticipate that the KS will be an acceptable way to optimise prescribing, increase their confidence and contribute to better antibiotic stewardship. The workshop and analysis strategy combined participative design with a sound theoretical approach to gauging intervention acceptability that can be used to benchmark future versions of the KS. This consistent monitoring approach could improve user focus in the process of developing eHealth tools.

## Supplementary Information

**Additional file 1:** **Supplementary file 1.** BRIT2 knowledge support system.

**Additional file 2:** **Supplementary file 2.** Topic guide.

**Additional file 3:** **Supplementary file 3.** Acceptability survey (Qualtrics).

**Additional file 4:** **Supplementary file 4.** Summary of raw data sources. **Table S4.1.** Quantitative data sources (data collated in Excel and IBM SPSS v25). **Table S4.2.** Qualitative data sources (uploaded to NVIVO 12+).

**Additional file 5:** **Supplementary file 5.** Suggestions to enhance intervention acceptability and user capability, opportunities and motivators. **Table S5.1.** Features to address clinician concerns (focal issues). **Table S5.2.** Suggestions to address clinician requirements. **Table S5.3.** Clinicians’ suggested features to include.

**Additional file 6:** **Supplementary file 6.** Application of workshop findings and visualisations.

## Data Availability

Data are available from the authors upon reasonable request and with permission of Prof. Tjeerd van Staa.

## Abbreviations

AB: Antibiotics
AI: Artificial Intelligence
AmR: Antimicrobial Resistance
BRIT Project: Building Rapid Interventions to optimise prescribing
CDSS/DSS: Clinical Decision Support System
CCG: Clinical Commissioning Group
GP: General Practitioner
KS: Knowledge Support System
NNT: Number Needed to Treat
Rx: Prescription
Sx: Symptoms
TFA: Theoretical Framework of Acceptability
