# Supplementary material for: Clinician acceptability of an antibiotic prescribing knowledge support system for primary care: a mixed-method evaluation of features and context
Source: BMC Health Serv Res. 2023 Apr 14;23:367. doi: 10.1186/s12913-023-09239-4 (PMC10103677; doi:10.1186/s12913-023-09239-4)
Supplement: Supplementary file 1 — Additional file 1: Supplementary file 1. BRIT2 knowledge support system. [file 12913_2023_9239_MOESM1_ESM.docx]

# Supplementary file 1 BRIT2 Knowledge Support System

The BRIT Knowledge Support System (KS) is an add-on piece of software that will integrate with prescriber electronic health record (EHR) systems [e.g. EMIS, TPP] for use during primary care consultations. The system aims to help clinicians to optimise the way they prescribe antibiotics by providing personalised analytics-based information about the patient to aid clinician decision-making. Initially the system will be restricted to adult patients (18-years and over) who are presenting with a common infection.

After diagnosis, the prescriber will trigger opening of the system (either manually via a button click or automatically triggered by EHR clinical codes or key words). The prescriber will enter answers to standard questions about the patient’s condition, symptoms and severity. Relevant patient data will then be extracted from the patient's EHR and evaluated in the context of clinical experiences of comparable patients as collected in large national research datasets, such as OpenSafely (1) and CPRD (2). In addition to a summary of pertinent history from the patient EHR's, the prescriber will then be presented with analytical information, including susceptibility to severe infection (if the patient were to go without antibiotics), risks related to potential antibiotic prescribing and alternative actions by other GPs. The prescriber will also be able to generate individualised patient leaflets to give to patients at the end of the consultation. Data from large national data sources will be used to get better understanding of the drivers for heterogeneity in care and for the clinical outcomes with different treatment strategies.

The KS is being developed as part of a Learning Health System in conjunction with other BRIT prescribing tools. Patient consultation data will be added into the system to inform the underlying model and future analyses. The results will provide baseline content for dashboards and reports that will be used to produce individual and practice level feedback to clinicians on their antibiotic prescribing patterns and outcomes. Clinicians will be supported to use the KS and give feedback to the developers using an online community of practice in an ongoing feedback loop between research and development and practical application for patient care.
