# Supplementary material for: Clinician acceptability of an antibiotic prescribing knowledge support system for primary care: a mixed-method evaluation of features and context
Source: BMC Health Serv Res. 2023 Apr 14;23:367. doi: 10.1186/s12913-023-09239-4 (PMC10103677; doi:10.1186/s12913-023-09239-4)
Supplement: Supplementary file 2 — Additional file 2: Supplementary file 2. Topic guide. [file 12913_2023_9239_MOESM2_ESM.docx]

# Supplementary file 2 Topic guide

Zoom poll1

**Part 1: Functionality focus group [Est. 50min]**

1.1 GROUP DISCUSSION Functionality (20min)

Padlet: Content for KS Features (Grade 1-10, Comment and Add Ideas)

1.2 GROUP DISCUSSION Opening the KS (15min)

Zoom Poll2 Opening the KS

1.3 GROUP DISCUSSION Data Credibility (10min)

Credibility Padlet suggestions (rating importance 1-10 + give option to add suggestions)

**Part 2 Patient Communication and Engagement [20min]**

2.1 GROUP DISCUSSION & Patient Communication Padlet

Zoom Poll 3 Patient Communication

**Part 3 Anticipated acceptability of the system: [45m]**

3.1 GROUP DISCUSSION acceptability & online questionnaire

Anticipated Acceptability Questions [focus group format]

Summing up/ Debrief [5 min]

Zoom poll4
