# Supplementary material for: Clinician acceptability of an antibiotic prescribing knowledge support system for primary care: a mixed-method evaluation of features and context
Source: BMC Health Serv Res. 2023 Apr 14;23:367. doi: 10.1186/s12913-023-09239-4 (PMC10103677; doi:10.1186/s12913-023-09239-4)
Supplement: Supplementary file 3 — Additional file 3: Supplementary file 3. Acceptability survey (Qualtrics). [file 12913_2023_9239_MOESM3_ESM.docx]

# Supplementary file 3 Acceptability survey (Qualtrics)

Prescriber Workshop Qualtrics Survey

A1a
**BRIT Knowledge Support System: Prescriber Workshop July 2021**
 
Please use this form to rate the acceptability of the BRIT Knowledge Support System, and tells us any thoughts you did not have chance to tell us in the workshop.

**1a. Affective Attitude - Rating**
On a scale of 0-10, how good or bad would you feel about using the Knowledge Support System?  (Drag the circle slider to rate)

|  | 0=Very bad, 10=Very good |
| --- | --- |

|  | 0 | 1 | 2 | 3 | 4 | 5 | 6 | 7 | 8 | 9 | 10 |
| --- | --- | --- | --- | --- | --- | --- | --- | --- | --- | --- | --- |

| Feeling () | 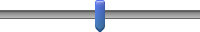 |
| --- | --- |

A1b
1b. Affective Attitude - Further Comments
How good or bad would you feel about using the Knowledge Support System?  
 
Consider:
Does the idea of using the Knowledge Support System evoke an emotional response?  If so which emotions are they? (E.g. stressed, worried, dismissive, pleased, enthusiastic) Is it a strong or weak feeling?

A2a
**2a. Burden - Rating**
On a scale of 0-10, how much effort do you think would be required to use the Knowledge Support System?  

|  | 0=No effort | 10=Lots of effort |
| --- | --- | --- |

A2b
**2b. Burden - Further Comments**
How much effort do you think would be required to use the Knowledge Support System?  

Consider: 
Examples of effort: time, expense, thought, memory. What aspects of using the Knowledge Support System do you think will require the most effort?

A3a
**3. Ethicality - Rating**
 On a scale of 0-10, how much is using the Knowledge Support System a good fit for your personal values?

|  |  |
| --- | --- |

A3b
**3b. Ethicality - Further Comments**
How much is using the Knowledge Support System a good fit for your personal values?

Consider:
What are your most important personal values? Would using the Knowledge Support System fit with those values? Are there any it would not fit with? Are there any moral/ethical issues in using the Knowledge Support System?

A4a
**4a. Self-efficacy - Rating**

on a scale of 0-10, how confident are you that you can use the Knowledge Support System to optimise your antibiotic prescribing?

|  | 0=Not at all | 10=Very |
| --- | --- | --- |

**4b. Self-efficacy - Further Comments**

How confident are you that you can use the Knowledge Support System to optimise your antibiotic prescribing?

Consider:
Feelings of control, confidence or capability to use the Knowledge Support System.

A5a **5a. Opportunity Costs - Rating**
On a scale of 0-10, to what extent might you give up any benefits, profits or values when you use the Knowledge Support System?

|  | 0=Not at all | 10=A lot |
| --- | --- | --- |

A5b **5b. Opportunity Costs - Further Comments**
To what extent might you give up any benefits, profits or values when you use the Knowledge Support System? 
Consider:
Time, for example, which you could have spent more profitably in the consultation. Any other things that you want to do / achieve that might interfere with using the KS

A6a **6a. Coherence - Rating**
On a scale of 0-10, how confident are you that you understand the Knowledge Support System and how it works?

|  | 0=Not at all | 10=Very |
| --- | --- | --- |

A6b **6b. Coherence - Further Comments**
How confident are you that you understand the Knowledge Support System and how it works?  
Consider: 
Is there anything that you need to help you understand the Knowledge Support System better?

A7a **7a. Perceived Effectiveness - Rating**

On a scale of 0-10, how confident are you that the Knowledge Support System is likely to optimise your antibiotic prescribing?

|  | 0=Not at all | 10=Very |
| --- | --- | --- |

A7b **7b. Perceived Effectiveness - Further Comments**

How confident are you that the Knowledge Support System is likely to optimise your antibiotic prescribing?
Consider: 
whether you feel the intervention will fulfil its aim; what factors influence this? E.g. past experience, your faith in the data, the session today.

I1 8a. How like is it that, given the opportunity, you will use the BRIT Knowledge Support System?

|  | Extremely Unlikely | Extremely Likely |
| --- | --- | --- |

|  | 0 | 1 | 2 | 3 | 4 | 5 | 6 | 7 | 8 | 9 | 10 |
| --- | --- | --- | --- | --- | --- | --- | --- | --- | --- | --- | --- |

I2 8b. **On a scale of 0-10, how much do agree/disagree with the following statements:**
'Assuming it is made available...

|  | I do not agree at all | I agree fully | (Not Applicable) |
| --- | --- | --- | --- |

|  | 0 | 1 | 2 | 3 | 4 | 5 | 6 | 7 | 8 | 9 | 10 |
| --- | --- | --- | --- | --- | --- | --- | --- | --- | --- | --- | --- |

| I intend to use the BRIT Knowledge Support System () | 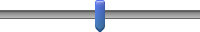 |
| --- | --- |
| I want to use the BRIT Knowledge Support System () | 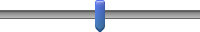 |

End of Block: Acceptability

Start of Block: Role

J1 **Job Role** - this information will help us understand which perspective the answers come from.
Choose your Job role 
(please select the closest option from the list below)

▼ GP (1) ... Other administrative role (9)

J2 Does your job role involve writing prescriptions?

▼ Yes (1) ... No (2)

E1 **Before you finish, we would like to ask some monitoring questions.**

These questions are optional but will help us to ensure that we are getting views from a wide range of people.

E2 Which one of the following best describes your gender?

▼ Female (1) ... Prefer not to say (4)

E3 Please select your age

▼ 18 (18) ... Prefer not to say (81)

E4 What is your ethnic group?

▼ White (1) ... Prefer not to say (6)
