# Supplementary material for: Clinician acceptability of an antibiotic prescribing knowledge support system for primary care: a mixed-method evaluation of features and context
Source: BMC Health Serv Res. 2023 Apr 14;23:367. doi: 10.1186/s12913-023-09239-4 (PMC10103677; doi:10.1186/s12913-023-09239-4)
Supplement: Supplementary file 4 — Additional file 4: Supplementary file 4. Summary of raw data sources. Table S4.1. Quantitative data sources (data collated in Excel and IBM SPSS v25). Table S4.2. Qualitative data sources (uploaded to NVIVO 12+). [file 12913_2023_9239_MOESM4_ESM.docx]

# Supplementary file 4 Summary of raw data sources

#### TABLE S4.1

Quantitative data sources (data collated in Excel and IBM SPSS v25)

| QUANTITATIVE DATA Sources | Number of  Examples/Questions | Number of  Responses |
| --- | --- | --- |
| *Padlet1Function Feature Ratings | 19 | 10 |
| *Padlet2Credibility Feature Ratings | 6 | 5 |
| *Padlet3Patient Communication Feature Ratings | 11 | 10 |
| Zoom Poll data 14 July | 5 | 9 |
| Zoom Poll data 21 July | 5 | 6 |
| Qualtrics survey (Acceptability / Intention) | 8 | 12 |
| Demographic data | 5 | 16 |

*Note*: *= Padlet totals include responses from workshops on both dates

#### TABLE S4.2

Qualitative data sources (uploaded to NVIVO 12+)

| QUALITATIVE DATA SOURCES | Code-able word count | Respondents |
| --- | --- | --- |
| SOURCE 1a - Audio transcription Files\\Recording_Audio_PW_14_July | 13188 (1078 from participant) | 4 |
| SOURCE 1b - Audio transcription Files\\Recording_Audio 21Jul | 11532 (2484 from participant) | 3 |
| SOURCE 2a - Zoom Files\\14Jul_Chat_Cleaned | 1456 (963 from participant) | 9 |
| SOURCE 2b - Zoom Files\\21Jul_chat_cleaned | 832 (656 from participant) | 7 |
| SOURCE 3 - TFA Survey\\Acceptability Survey Qualitative Results (comments) | 1100 | 12 |
| SOURCE 4 - Feedback Email | 307 | 1 |
| SOURCE 5 - Padlet comments | 91 | NA  [7comments] |
