# Supplementary material for: Clinician acceptability of an antibiotic prescribing knowledge support system for primary care: a mixed-method evaluation of features and context
Source: BMC Health Serv Res. 2023 Apr 14;23:367. doi: 10.1186/s12913-023-09239-4 (PMC10103677; doi:10.1186/s12913-023-09239-4)
Supplement: Supplementary file 5 — Additional file 5: Supplementary file 5. Suggestions to enhance intervention acceptability and user capability, opportunities and motivators. Table S5.1. Features to address clinician concerns (focal issues). Table S5.2. Suggestions to address clinician requirements. Table S5.3. Clinicians’ suggested features to include. [file 12913_2023_9239_MOESM5_ESM.docx]

# Supplementary file 5 Suggestions to enhance intervention acceptability and user capability, opportunities and motivators.

Suggestions for design and implementation measures to address the themes highlighted by clinicians in the qualitative analysis. For each suggestion the dimension of intervention acceptability [TFA, (3)] and user behaviour components [COM-B (4)] are noted with the source of the insights.

TABLE S5.1 Features to address clinician concerns (focal issues)

| **Workshop**  **Themes** | **Suggestions to address clinicians’ concerns** (focal issues) | **TFA Acceptability**  **Dimensions**  **Addressed** | **COM-B** | **Suggestion based on** | | | | | |
| --- | --- | --- | --- | --- | --- | --- | --- | --- | --- |
|  |  |  |  | **TA** | **ZP** | **P** | **AS** | **D** | **PP** |
| **Concern**:  Safe and accurate prescribing | (E)Ensure the KS doesn’t override safety protocols built into the EHR e.g. letting the prescriber select a contraindicated medication [general safety] | Eth, Aff | M |  |  |  |  |  |  |
|  | (E) Flag warning symptoms for severe infection | Eth, Aff, SeE | M,PO | / |  | / |  |  |  |
|  | (E) Personalised patient risk information | Eth, Aff, OpC | M,PO,SO,PC |  |  | / |  |  |  |
|  | Be able to suggest antibiotics (suitable for that individual) and an alternative | PeE, OpC, SeE | PC,PO | / |  |  |  |  |  |
|  | Include recommended actions to flag patients who are vulnerable to infection/ AmR | Eth, PEf, OpC, Bur, SeE | PO,M |  |  | / |  |  |  |
| **Concern**: Accessibility/ Support user’s cognitive processing | (E) Don’t force clinician to rely on memory (e.g. transferring data from one page to another) | Bur, PEf | PsC | / |  |  | / | / |  |
|  | (E) Autocomplete to speed up completion (e.g. name of antibiotics) | Bur, PEf | PO | / |  |  |  |  |  |
|  | (E) Use logical grouping to display information that is relevant to the prescribing decision | Coh | PO | / |  |  |  |  |  |
|  | (E) Ensure it is clear what the risk statistics are indicating | Eth, Coh | PO,PsC | / |  |  |  |  |  |
|  | (E) Show patient communication options (if possible allow clinician to easily check that those details are up-to-date) | Bur, Coh | PO |  | / | / |  |  |  |
|  | (E) Include prompts for |  |  |  |  |  |  |  |  |
|  | -Factors that might form the basis of a patient conversation (this might be prompted in the selections for the Patient information) | SeE, Aff | PsC, SO,M | / |  |  |  | / |  |
|  | -Key points about the treatment decision to discuss with the patient | SeE, Aff | PsC, SO, M | / |  |  |  | / |  |
|  | Colour coding for fast recognition e.g. of important risk factors | Bur, Coh | PsC,PO |  |  |  |  |  |  |
| **Concern**:  Autonomy | Consider that prescribers will want to work in different ways (depending on experience) and build flexibility into the tool (e.g. order of data completion/ being able to dip into the tool to check information) | Bur, Coh, SEff, Aff | PsC,PO,M | **/** |  |  |  |  |  |
|  | (E) Give clinician the opportunity to change their mind e.g. change diagnosis | Bur, SEff, PEf | PSc, M | **/** |  |  |  |  |  |
|  | (E) Opportunity to customise notes written back to the EHR | Bur, SEff, Aff | PO |  |  |  | **/** |  |  |
|  | Personalise settings e.g. choose how the KS opens/ appearance/ display | Bur, Aff | M | **/** |  |  |  |  |  |
|  | Option to add information to the patient communication | PEf | PO, M |  |  |  |  | **/** |  |

*Notes*: **Source of Suggestion:** TA = Thematic Analysis; ZP= Zoom Polls; P= Padlets; AS= Acceptability; D= Discussion with GP Consultant; PP=PPIE group meeting

**TFA Dimensions feature aims to address:** Aff= emotional acceptability; Bur = reduce burdens; Eth=Fit ethical values; SEff = feelings that clinician is performing effectively; OpC = reduce opportunity costs; Coh = increase user understanding; PEf = meet expectations of effective performance.

**[COM-B Factors this would help address to optimising prescribing**: M = motivation; SO = Social Opportunities; PO = Physical Opportunities; PsC = Psychological Capabilities

TABLE S5.2 Suggestions to address clinician requirements

| **Workshop**  **Theme** | **Suggestions to address clinician Requirements** | **TFA Dimension Of Acceptability** | **COM-B** | Suggestion based on | | | | | |
| --- | --- | --- | --- | --- | --- | --- | --- | --- | --- |
|  |  |  |  | **TA** | **ZP** | **P** | **AS** | **D** | **PP** |
| **Requirement**: Easy and efficient to use | (E) Simple intuitive design which allows easy access to a range of patient information to support the prescribing decision | Bur, Coh, SeE | PO | / |  |  | / | / |  |
|  | (E) The system should load and process quickly – single point sign in with EHR would be ideal | Bur, OpC, Coh | PO | / |  |  |  |  |  |
|  | (E) Minimise data input requirements and maximise assistance with logging the decision and consultation outcomes. | Bur, PEf, OpC | PO | / |  | / | / |  |  |
|  | Ability to open the KS from the EHR tool bar | Bur, Coh | PO | / | / |  |  | / |  |
|  | (E) Ability to have access/view concurrently with EHR (no conflict) | Bur, Coh, PEf | PO,PsC | / |  |  | / |  |  |
|  | Allow clinician to save progress or return to information (ease of use). | Bur, Coh | PO | / |  |  |  |  |  |
| **Requirement**:  IT Confidence/ Training an documentation | (E) Have a training session with prescribers (at a minimum train a representative at each surgery) | SeE, Coh, Aff, Bur | PsC, M | **/** |  |  |  |  |  |
|  | (E) Have a procedure in place for help queries/technical issues | SeE, Coh, Aff, Bur | PsC, M | **/** |  |  |  |  |  |
|  | Automatic help E.g. login reset | SeE, Coh, Aff, OpC | PO, M | **/** |  |  |  |  |  |
|  | FAQ on the OCoP | SeE, Coh, Aff, Bur | PO, SO, M |  |  |  |  |  |  |
|  | Peer support via the OCoP | SeE, Coh, Aff, Bur | SO, M |  |  |  |  |  |  |
|  | Work with prescribers to outline realistic procedures to follow for common tasks ensuring the KS fits with work flows (guidelines) | Coh, Bur, OpC, PEf | PO, PsC | **/** |  |  | **/** |  |  |
|  | Have a procedure for those who are having more difficulties than average using the KS. | SeE, Aff, OpC | PO, PsC, SO, M | **/** |  |  | **/** |  |  |
|  | Have a procedure for those who are not receptive to using the KS (e.g. one to one discussion with a researcher). | SeE, Aff, OpC | PO, PsC, SO, M | **/** |  |  | **/** |  |  |
| **Requirement**  Clarity/ Perceived Efficacy | (E) You should be able to click or hover over risk scores to see what variables were included (at minimum this information should be available on a separate page or outside the system) | Coh, PEf, Eth | PO, PsC | **/** |  | **/** |  |  |  |
|  | (E) A video and or literature (with an expert ideally) explaining the system clarifying what it does and how it is helpful for 1)Safe prescribing, system and data checking 2)Efficient working 3)Patient Outcomes 4)Technical support provision | PEf, Coh, Eth | PsC,SO, M | **/** |  |  | **/** |  |  |

*Notes*: **Source of Suggestion:** TA = Thematic Analysis; ZP= Zoom Polls; P= Padlets; AS= Acceptability; D= Discussion with GP Consultant; PP=PPIE group meeting

**TFA Dimensions feature aims to address:** Aff= emotional acceptability; Bur = reduce burdens; Eth=Fit ethical values; SEff = feelings that clinician is performing effectively; OpC = reduce opportunity costs; Coh = increase user understanding; PEf = meet expectations of effective performance.

**[COM-B Factors this would help address to optimising prescribing**: M = motivation; SO = Social Opportunities; PO = Physical Opportunities; PsC = Psychological Capabilities

TABLE S5.3 Clinicians’ suggested features to include.

| **Workshop**  **Theme** | **Clinicians’**  **Suggested Features to include in the KS and Patient Leaflet** | **TFA Dimension Of Acceptability** | **COM-B** | Suggestion based on | | | | | |
| --- | --- | --- | --- | --- | --- | --- | --- | --- | --- |
|  |  |  |  | **TA** | **ZP** | **P** | **AS** | **D** | **PP** |
| **Features:**  Input | (E) Minimise data input (extract information from EHR wherever possible) | Bur, PEf, | PO | **/** |  |  | **/** |  |  |
|  | (E) Manual opening | Bur, PEf, Aff | M | **/** | **/** |  |  |  |  |
| **Features**: Output | (E) Summary of information relevant to whether to prescribe antibiotics  Include: relevant patient conditions, previous antibiotics, previous hospitalisation, and suitable antibiotics. Re: treatment recommendation: suitable antibiotics and formulations that considered interactions with existing medication and co-morbid conditions, back-up options, dose adjustments, creatinine, weight, age, gender | PEf, Eth | PO, PsC | **/** |  |  |  |  |  |
|  | Recommended Actions  Include: personalised treatment recommendation, What treatment/action to try if treatment fails, and warning symptoms for patient referrals. | SEf, PEf, Aff | PO, PsC | **/** |  | **/** |  |  |  |
|  | (E) Add logo of funders , UoM/ Brit/ NICE/ NHS | PEf, Eth | SO, M |  |  | **/** |  |  |  |
|  | Functionality to upload the CCG and or Practice logo | PEf, Eth | SO, M |  |  | **/** |  |  |  |
|  | Patient Risk Indicators  Include: Risk scores; systemic infection, infection-related hospitalisation, serious side-effects, personalised risk factors | SEf, PEf, Aff | PO,PsC | **/** |  | **/** |  |  |  |
|  | Reminders/Caution flags  Include: Patient AmR risk, local AmR issues, and common actions taken by peers with similar patients. | SEf, PEf, Aff | PO, M | **/** |  | **/** |  |  |  |
| **Features**: Patient Communication | (E) PDF or web-based output that is able to be printed or sent out via a text link | PEf, Eth | PsC, PO | **/** |  | **/** |  | **/** |  |
|  | (E) Resources to support the clinicians’ discussion with the patient | SeE | PsC, PO,SO | **/** |  | **/** |  |  |  |
|  | Personalised patient vulnerabilities (to infection/AmR) and potential side-effects especially where a patient has had repeat antibiotic use. | Eth | M |  |  |  |  |  |  |
|  | Option to output a version in an alternative language (such as Polish, Urdu, Guajarati) | Aff, Eth, Bur | PsC, PO | **/** |  | **/** |  | **/** |  |
|  | Option to send out a large font version (font 24) | Aff, Eth, Bur | PsC, PO |  |  |  |  |  |  |
|  | Justification for the treatment decision | Bur, PEf | PsC, PO |  |  | **/** |  |  | **/** |
|  | Include information about the number of times the patient has had antibiotics | Coh | PsC, PO | **/** |  | **/** |  |  | **/** |
|  | Make any risk information very simple and intuitive to understand | Bur, Coh | PsC | **/** |  |  |  |  | **/** |
|  | Warning signs of serious infection and urgent contact details | Eth, SeE, Coh | PO | **/** |  | **/** |  | **/** | **/** |
|  | How many days until you should contact the surgery again | Eth, SeE, | PsC |  |  |  |  | **/** |  |

*Notes*: **Source of Suggestion:** TA = Thematic Analysis; ZP= Zoom Polls; P= Padlets; AS= Acceptability; D= Discussion with GP Consultant; PP=PPIE group meeting

**TFA Dimensions feature aims to address:** Aff= emotional acceptability; Bur = reduce burdens; Eth=Fit ethical values; SEff = feelings that clinician is performing effectively; OpC = reduce opportunity costs; Coh = increase user understanding; PEf = meet expectations of effective performance.

**[COM-B Factors this would help address to optimising prescribing**: M = motivation; SO = Social Opportunities; PO = Physical Opportunities; PsC = Psychological Capabilities
