# Supplementary material for: Clinician acceptability of an antibiotic prescribing knowledge support system for primary care: a mixed-method evaluation of features and context
Source: BMC Health Serv Res. 2023 Apr 14;23:367. doi: 10.1186/s12913-023-09239-4 (PMC10103677; doi:10.1186/s12913-023-09239-4)
Supplement: Supplementary file 6 — Additional file 6: Supplementary file 6. Application of workshop findings and visualisations. [file 12913_2023_9239_MOESM6_ESM.docx]

# Supplementary file 6 Application of workshop findings and visualisations

## 6.1 Overview of KS design implications

Clinician inputs to the KS should be extracted from the EHR wherever possible for time, accuracy, to reduce cognitive load and to assist users with learning difficulties. The presentation of the outputs needed to be brought together to support cognition (memory and attention) which would aid clinician’s holistic decision-making, reduce time searching for details, avoid key details being missed and help clinicians spot opportunities for patient discussion / education more easily. Although clinicians wanted to be in control of when to use the system, they did want to see recommended actions, particularly in circumstances where the patient was at high risk of systemic infection, when delayed antibiotic prescribing was appropriate, and actions to ‘try next’ when patients re-consult (for example because the previous antibiotic did not work). Selection of a safe and appropriate antibiotic (where indicated) was high priority concern and could be extremely complex for some patients. Several participants felt personalised treatment suggestions would improve confidence, reduce the need to check with senior colleagues (or deferring to other source material), and make prescribing more effective for the patient. The research team’s commitment to user-focus and patient-care was also important to clinicians (as indicated concerns and values identified) and maintaining this focus during ongoing development of the KS is important.

Prominent themes of the workshop and clinician suggestions were tabulated as recommendations and features that would help make the KS more likely to be used by increasing acceptability (see *Supplementary File 5*). As the main aim of the KS is to optimise antibiotic prescribing behaviour, annotations suggesting relevant areas of behaviour change using the Capability Opportunity Motivation framework COM-B (5). The majority of the recommendations would support antibiotic prescribing by increasing physical opportunities (such as information and prompts), supporting clinicians’ psychological capabilities (such as cognitive processes and knowledge) with some increased motivation and social opportunities to address patient behaviour. Visualisations of the user interface (Figure S6.1) and patient leaflets (Figure S6.2) were created to help summarise and communicate desired features to the intervention development team.

## 6.2 KS visualisations

## FIGURE S6.1 User interface Summary page

AB and or Infection history with the option to search back further

(Relevant information to assist AB decision making grouped together)

Flag for recent hospital admissions


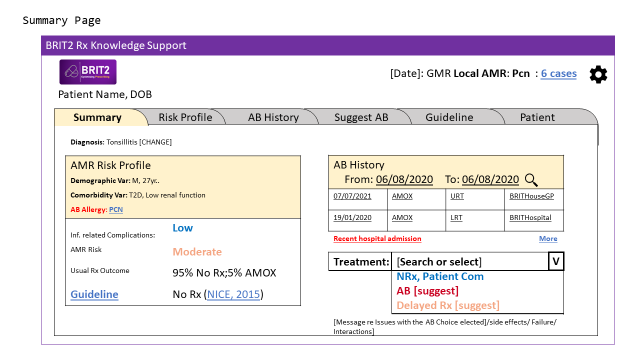


Link to guideline and /or an indication of what the guideline suggests

AB/ AmR Risk information

Ability to search or select AB without moving away from this screen

Flags/ patient conditions/ levels to consider

## FIGURE S6.2 Patient leaflet visualisation


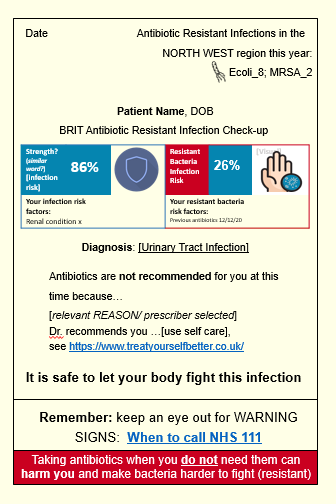
 (No Antibiotics) – Visualisation with suggested features

Image of bacteria on the body (personal relevance)

Visual (e.g. shield) and numeric indicator of risk (reframed as a strength)

Reminder of diagnosis

Relevant factors (patient education/ awareness)

Sign posting (first port of call for advice)

Reassurance that it is safe not to have AB

Messaging to reframe taking AB when not needed as risky/ potentially harmful

Reasoning (help GP explain)

Link to recommended self-care treatment

Link to infection warning signs to look out for

Colour coding (accessible colours)

Inform about local AMR (relevance)

1. OpenSAFELY. Secure analytics platform for NHS electronic health records Online: Bennett Institute for Applied Data Science; 2022 [Available from: <https://www.opensafely.org/>.

2. NIHR, MHPRA. Clinical Practice Research Datalink: Medicines and Healthcare products Regulatory Agency.,

National Institute for Health and Care Research (NIHR).,; 2022 [Available from: <https://cprd.com/>.

3. Sekhon M, Cartwright M, Francis JJ. Acceptability of healthcare interventions: an overview of reviews and development of a theoretical framework. BMC Health Serv Res. 2017;17(1):88.

4. Michie S, van Stralen MM, West R. The behaviour change wheel: a new method for characterising and designing behaviour change interventions. Implement Sci. 2011;6:42.

5. Michie S, Atkins L, West R. Behaviour Change Wheel: A Guide to Designing Interventions: Silverback Publishing; 2018. Available from: <https://r3.vlereader.com/Reader?ean=9781912141081>.
